# Construction Jobsite Image Classification Using an Edge Computing Framework

**DOI:** 10.3390/s24206603

**Published:** 2024-10-13

**Authors:** Gongfan Chen, Abdullah Alsharef, Edward Jaselskis

**Affiliations:** 1 Department of Civil, Construction and Environmental Engineering, North Carolina State University, Raleigh, NC 27695, USA; ejjasels@ncsu.edu; 2 Civil Engineering Department, King Saud University, Riyadh 11451, Saudi Arabia; aalsharef@ksu.edu.sa

**Keywords:** edge computing, construction image classification, quantization, transfer learning, Raspberry Pi, Edge TPU, material classification, safety detection

## Abstract

Image classification is increasingly being utilized on construction sites to automate project monitoring, driven by advancements in reality-capture technologies and artificial intelligence (AI). Deploying real-time applications remains a challenge due to the limited computing resources available on-site, particularly on remote construction sites that have limited telecommunication support or access due to high signal attenuation within a structure. To address this issue, this research proposes an efficient edge-computing-enabled image classification framework for support of real-time construction AI applications. A lightweight binary image classifier was developed using MobileNet transfer learning, followed by a quantization process to reduce model size while maintaining accuracy. A complete edge computing hardware module, including components like Raspberry Pi, Edge TPU, and battery, was assembled, and a multimodal software module (incorporating visual, textual, and audio data) was integrated into the edge computing environment to enable an intelligent image classification system. Two practical case studies involving material classification and safety detection were deployed to demonstrate the effectiveness of the proposed framework. The results demonstrated the developed prototype successfully synchronized multimodal mechanisms and achieved zero latency in differentiating materials and identifying hazardous nails without any internet connectivity. Construction managers can leverage the developed prototype to facilitate centralized management efforts without compromising accuracy or extra investment in computing resources. This research paves the way for edge “intelligence” to be enabled for future construction job sites and promote real-time human-technology interactions without the need for high-speed internet.

## 1. Introduction

With rapid digital transformation in the construction industry, various reality-capture technologies are being implemented on construction sites for data collection [1]. Prominently, construction images are frequently captured using smartphones [2,3], drones [4,5], surveillance cameras [6,7], and unmanned ground vehicles (UGV) systems [8]. These images provide valuable information that can facilitate automated monitoring, inspection, and tracking processes. Researchers often utilize these images to identify safety hazards [9,10,11], monitor workers’ productivity [12,13], track resources [14,15,16], and facilitate progress monitoring [17].

One of the most widely used methods in image analysis involves applying artificial intelligence (AI) models for image classification. To create a customized classification system, these images are often labeled based on precise criteria tailored to specific needs. Deep learning models, especially convolutional neural networks (CNNs), are commonly used to identify patterns and extract features from these images [18]. Through iterative training, these models enhance their ability to accurately classify new, unseen images. For real-time applications, the well-trained models will be considered to be embedded into on-site devices such as surveillance cameras [9], mobile phones [19], and drones [11,20] to run inferences with the support of the internet. However, these devices are generally battery-powered and constrained by limited computational capabilities [21,22]. Construction structures with heavy reinforcement materials, such as steel or reinforced concrete, can also significantly affect internet connectivity by causing signal attenuation and interference [23]. As a result, obtaining sufficient computing resources to deploy this real-time application is particularly challenging, making real-time implementation still far from reality, especially in rural construction areas where such resources are often scarce.

Recently, edge computing has become popular as a distributed computing diagram, where data processing and analysis take place in proximity to the data source or the “edge” of the network [24,25]. This computing framework supports real-time image capture and processing, allowing for model inference to be performed locally using the computing power of edge devices. As the demand for more real-time analytics and process intelligence grows, this approach significantly reduces latency in real-time responses and optimizes bandwidth usage [24,25]. With advancements in microchip technology, the possibility of both training and running inference directly on edge devices is becoming increasingly feasible [26]. Currently, image-based edge computing solutions involve training models in the cloud and then deploying the trained models to edge devices like Raspberry Pi [27], NVIDIA’s Jetson series [28], and Arduino [29], with supports by battery modules [22], and other edge servers [30,31]. The NVIDIA Jetson series is specifically designed for machine learning (ML) and computer vision tasks [32]. However, these methods primarily depend on the computing power of these edge devices. As engineering problems grow in complexity, it is necessary to develop a generalized framework that can optimize the utilization of computing resources regardless of the sophistication of image classification models. Researchers emphasized that construction companies should focus on deployment strategies rather than the specific tools themselves when considering investment in computing resources [33].

This research follows a fundamental engineering research design methodology elaborated by Hazelrigg [34] to apply an edge computing-enabled efficient image classification framework for real-time construction job site applications. The specific objectives are to (1) create a lightweight binary image classification model tailored for practical construction applications; (2) investigate and optimize the deployment of construction image classification tasks on the edge; and (3) integrate a complete edge computing environment to facilitate real-time implementation of image classification in construction settings. To achieve these goals, the essential steps are as follows: (1) This research employed transfer learning (TF) to develop a lightweight image classification model based on MobileNet. (2) The model was then optimized through quantization, reducing its size while preserving inference accuracy using the TensorFlow Lite (TFLite) framework. This process ensured efficient deployment on edge devices. (3) Finally, a real-time implementation system was assembled, consisting of a Raspberry Pi (single-board computer), PiCamera, Edge TPU (accelerator), and other necessary hardware and software components. (4) Two case studies, including construction material classification and construction nail detection, are implemented in real time in practice to validate the research framework.

## 2. Literature Review

### 2.1. Edge Computing

Edge computing processes data closer to its sources by leveraging local computing resources, such as Internet of Things (IoT) devices, leading to faster data insights, improved response times, and more efficient bandwidth utilization [24]. This method improves computational efficiency, reduces the use of signal transmission channels, eases the storage and processing demands on cloud servers, and facilitates real-time data analysis and decision-making close to the data source [24,35]. Cloud computing encounters several significant challenges due to its centralized nature. As a solution, Edge Computing has been introduced to enhance performance and address cloud-related issues by enabling local data processing and storage at the end devices [36]. The rapid evolution of edge computing is powered by the increased computing capabilities of microchips, where the quantity of transistors on a microchip doubles every 18 months while decreasing costs by half according to Moore’s Law [37]. Edge computing began in the 1990s with the concept of content delivery network (CDN), describing a network model that placed computing nodes closer to users for faster-cached content (e.g., images, videos) delivery [38]. In 1997, Nobel et al. explored how applications (web browsers, video, and speech recognition) on mobile devices could shift some processing tasks to powerful local servers, reducing the burden on resource-constrained devices and enhancing the battery life of mobile devices [39]. This concept was generalized as “pervasive computing” later in 2001 [40]. At the same time, Rowstron and Druschel introduced distributed hash tables that utilized the proximity of physical internet connections for efficient data routing and load balancing to improve communication latency and reduce network load [41]. As more and more computing and storage resources are needed, cloud computing emerged as a major influencer for edge computing in 2006 [42]. Researchers also designed a “cloudlet” to provide nearby mobile devices with computing and storage resources [43]. By 2012, fog computing emerged as a solution to the growing demand for scalable IoT infrastructures, enabling the management of vast numbers of devices and the processing of large volumes of data [25,44].

The earliest work on edge computing concepts in construction dates back to 1985, when researchers aimed to leverage small computers for various construction planning and execution tasks [45]. In the past few years, mobile computing has been widely adopted to enable real-time applications in the construction fields [19,46,47,48]. It involves bringing mobile devices with wireless connectivity to construction sites for real-time information sharing [49]. For example, researchers have combined mobile computing with Augmented Reality (AR) and wireless communication technologies for real-time site monitoring, task management, and information dissemination [19,46,50]. With the advent of building information modeling (BIM), various applications built upon mobile computing have been developed to streamline BIM data collection and communication both on and off construction sites [51,52,53]. However, mobile computing heavily relies on centralized cloud services for network connectivity and data transmission [51,54], which may be problematic in construction scenarios located in regions with limited or no network coverage. The term “edge computing” was first coined in the construction industry in 2018, when Kochovski et al. [21] developed an edge computing infrastructure designed to deliver high Quality of Service (QoS) for video communications and construction process documentation, addressing the growing demand for computing resources on construction sites. As data volumes continue to grow exponentially nowadays, researchers have started using AI models for rapidly processing and extracting valuable insights from different data sources to drive decision-making. The increasing analytical demands of these models have prompted researchers to explore solutions that offload specific computational tasks to edge servers [30,31]. Consequently, AI models are being embedded into edge devices for real-time applications, such as safety detection [55], resource tracking [28], and structural health monitoring [56,57]. The integration of edge computing into construction fields represents significant opportunities to reduce latency in data communications for time-sensitive tasks [55], improve data privacy and security through local processing [58,59], and optimize network bandwidth usage by transmitting only relevant information [22,60]. Overall, the integration of IoT and edge computing on construction sites offers powerful solutions to enhance productivity, improve quality, and boost safety, making these technologies essential for advancing efficiency and innovation in the construction industry [61].

**Figure 1:**
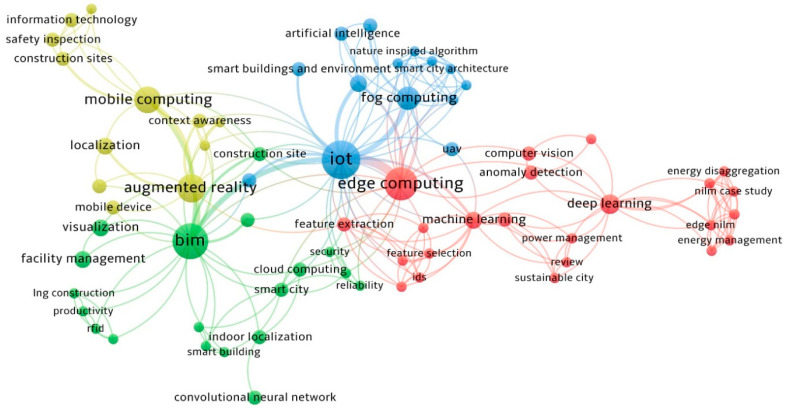
Co-occurrence of trending research topics.

**Figure 2:**
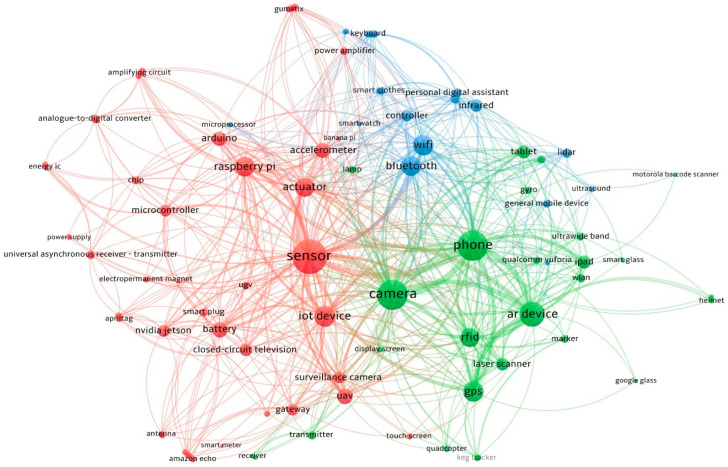
Co-occurrence map of the implemented device/hardware.

To gain a high-level understanding of mobile edge computing research trends in the architecture, engineering, and construction (AEC) industry, 106 relevant research articles were pulled from the Web of Science (WoS) database between 2010 and 2023 to draw two co-occurrence diagrams: one focused on mobile edge computing keywords, one focused on implemented devices/hardware for mobile edge computing, as shown in Figure 1 and Figure 2. Figure 1 indicates that mobile computing research is often related to augmented reality and BIM, including research topics of localization [62], facility management [63], context awareness computing [64,65], and visualization [66,67]. Edge computing is closely associated with IoT, ML, and deep learning, highlighting emerging trends in developing analytic capabilities in edge computing. The integration of edge computing and AI techniques significantly contributes to advancements in research areas, including smart buildings [68], smart cities [69,70], anomaly detection [56,71], and energy management [60]. Figure 2 highlights the sensors, which are the core technology for mobile/edge computing. They are connected with various mobile computing devices, such as phones and cameras. These devices are extensively connected with mobile connectivity technologies (Wi-Fi, Bluetooth) [22,72], AR, [66,73,74], and location systems (GPS, RFID) [51,75], highlighting potentials integration in practices such as safety, navigation, and user interfaces. Edge computing mainly requires a single board of a computer, such as Raspberry Pi [27,28] and Arduino [29], for edge computing capabilities. Battery is another factor that allows edge devices to operate independently and continuously with a power supply [22]. Actuators are also crucial because they enable edge devices to interact directly with and control physical systems in real time for immediate response and automation [21]. Nonetheless, this research organized 106 reviewed documents and put them in a shared spreadsheet, which can be found at the Supplementary Materials in the paper.

### 2.2. Image Classification

Image classification has been a fundamental task in computer vision, aiming to categorize images into predefined classes using supervised ML. The early approaches to image classification relied heavily on hand-crafted features such as Scale-Invariant Feature Transform (SIFT) and Histogram of Oriented Gradients (HOG), combined with traditional ML models like Support Vector Machines (SVM) and Random Forests [76,77]. However, the advent of deep learning, particularly CNNs, revolutionized the field [78]. CNNs are designed to automatically learn hierarchical feature representations from raw images, which significantly reduces the need for manual feature extraction. Pioneering models like AlexNet [79], VGGNet [80], and ResNet [81] demonstrated remarkable improvements in image classification accuracy on large-scale datasets such as ImageNet [82]. These models leverage multiple layers of convolutional filters to capture spatial hierarchies in images, thereby achieving state-of-the-art performance. Recent advances in image classification have focused on enhancing the efficiency and accuracy of CNNs through various approaches. Techniques such as TF and data augmentation have become standard practices to improve model generalization, particularly in scenarios with limited labeled data [83]. TF involves pre-training a CNN on a large dataset and fine-tuning it on a target task, allowing for faster convergence and better performance. Additionally, the development of attention mechanisms, such as the Transformer-based Vision Transformer (ViT), has further advanced image classification by enabling models to focus on relevant parts of an image, mimicking human visual attention [84]. These innovations continue to push the boundaries of image classification within the field of computer vision.

In recent years, there has been a shift towards optimizing model architecture for deployment in resource-constrained environments, such as edge devices. This has led to the development of lightweight models like MobileNet [85], ShuffleNet [86], and EfficientNet [87], which prioritize a balance between accuracy and efficiency. MobileNet and ShuffleNet utilize depth-wise separable convolutions and group convolutions, respectively, to reduce the model size and computational load, allowing for faster inference speeds [88]. EfficientNet introduces a compound scaling approach that systematically scales the depth, width, and resolution of the network, achieving state-of-the-art performance with fewer parameters [87]. These models have been instrumental in enabling real-time image classification on devices with limited processing power and memory. For example, Raza et al. [89] trained an EfficientNet model that utilized less computational resources and training time while maintaining high accuracy in classifying lung cancer using CT scan images with a performance score between 0.97 and 0.99 on the test set. As the demand for efficient and accurate models continues to grow, further research is focused on developing architectures that maintain high performance while minimizing complexity and resource consumption.

### 2.3. Construction Site Image Classification Applications

The use of image classification in construction has seen significant growth, particularly in automating the monitoring and management of construction sites [90,91]. By leveraging computer vision technologies, construction companies can analyze real-time images captured from various sources, such as drones [4,5], fixed cameras [17,92], and mobile devices [2,3]. These images are utilized to track progress [93,94], ensure safety compliance [9,95], defect detection [96,97], and monitor the usage of materials and equipment on-site [98,99]. It has been identified that CNNs are the most widely used models to perform classification tasks [18]. These image classification applications can significantly reduce the management burden on central managers, allowing them to focus more on strategic problem-solving rather than time-consuming manual inspections [94].

Despite the promising potential of image classification in construction, several limitations hinder its widespread adoption. The primary challenge is the scarcity of annotated datasets tailored specifically to construction environments, which hampers effective model development [18]. One promising solution is TF, where models are pre-trained on large datasets that can be fine-tuned on smaller, domain-specific datasets, thereby reducing the need for extensive annotations. Researchers have shown that TL can improve the ability of image classification models to learn fundamental data patterns, often resulting in better results [3]. Another major limitation is the computational cost and the demand for high-performance hardware to process images in real time. As engineering problems grow more complex, image classification models tend to scale up because of more parameters, requiring additional computing resources for efficient inference [85]. Running these models on construction sites using devices such as smartphones, AR/VR tools (e.g., HoloLens, Daydream), and drones is challenging due to the limited memory and computing power of these devices [100], making it still far from reality to implement these models in real-time.

The integration of edge computing and image classification models presents significant potential for real-time applications on construction sites. For example, Tan et al. [20] embedded a crack detection model into an unmanned aerial vehicle (UAV), enabling real-time synchronization of crack data collection and analysis on building surfaces. The model was executed directly on DJI’s Onboard SDK (OSDK), a development kit designed for edge computing. Similarly, Chen et al. [28] integrated a construction hardhat detection model into a Raspberry Pi, facilitating real-time video data processing for improved worker management. Another study evaluated a worker detection model on both a local computer and three edge computing devices (Jetson Nano, Raspberry Pi 4B, and Jetson Xavier NX). The results demonstrated that while all devices achieved comparable accuracy, the local computer exhibited the fastest processing speed, followed by the Jetson Xavier NX, with the Raspberry Pi 4B being the slowest [101]. Additionally, the Raspberry Pi 4B consumed the most CPU resources due to its lack of a GPU. Based on the information provided, these studies mainly depend on the inherent computing capabilities of edge devices without optimizing the implementation frameworks. As more advanced image classification models are deployed, issues like inference speed and memory usage become critical, making the previous approach difficult to generalize. For instance, the simplest ViT-Base model [84], which has 86 million parameters, is significantly larger than widely used CNN architectures like ResNet-50 [81], which has 25 million parameters. This disparity highlights the necessity of developing a framework that optimizes computing resource utilization for a more generalized real-time image classification application in construction sites, especially in resource-constrained environments like rural construction sites.

## 3. Methodology

### 3.1. Overview

**Figure 3:**
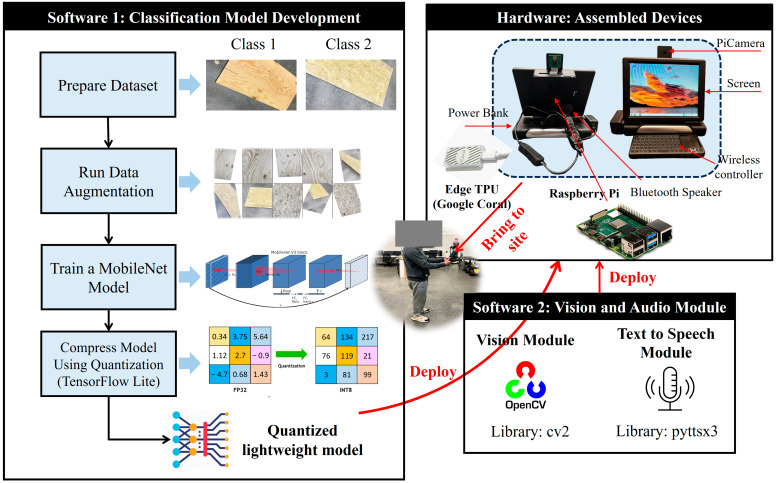
Edge computing implementation framework.

This study employs the fundamental engineering research design methodology as delineated by Hazelrigg [34]. Rather than aiming to optimize any existing models, the focus is on implementing an integrated edge computing framework for image classification tasks within construction job site environments. Figure 3 illustrates the research framework and the developed hardware module. The primary aim of this research is to explore binary classification techniques for various real-time construction applications. After determining the specific use case and collecting the necessary data, this research was initiated by data augmentation, such as flipping, rotating, and shearing to the original images, thereby increasing the dataset size. We then employed a MobileNet TF to train a lightweight binary classifier, enabling it to distinguish between two distinct classes and evaluate the performance using various model performance metrics. This lightweight model can largely save computing resources when running inference. The resulting model was quantized using TFLite to further reduce its size for deployment on edge devices. We conducted a detailed analysis of the trade-offs between accuracy and model size across different models. Additionally, this research developed a custom Raspberry Pi hardware module equipped with a screen, PiCamera, power bank, Bluetooth speaker, wireless keyboard, touchpad mouse, and accelerator coprocessor. Finally, we integrated a visual, textual and audio module into the edge device to facilitate a synchronized real-time interactive multimodal detection system.

### 3.2. Classification Model Development

As a preliminary examination, this study evaluated traditional convolutional neural network (CNN) models and various fine-tuning methods (e.g., change activation function, batch normalization, and data preprocessing). However, the initial results indicated that CNN models built from scratch underperformed and exhibited suboptimal model sizes. In contrast, when testing pre-trained models, particularly lightweight MobileNet architectures, we found a significant increase in accuracy without compromising model size. This initial outcome directed the research efforts toward the development and utilization of these lightweight models.

**Figure 4:**
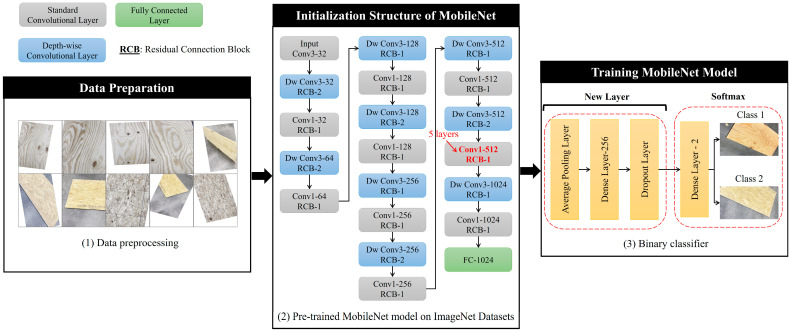
MobileNet architecture and model development process.

MobileNet is a lightweight and efficient CNN architecture featured on depth-wise separable convolutions, including depth-wise convolutions and point-wise convolutions, allowing it to achieve a good balance between model size and performance [102,103]. The model architecture and development process are shown in Figure 4. In this study, we leveraged TL to deploy the MobileNet model and compared different versions of MobileNet to select the optimal version. Note that the current training process under the edge computing framework is still in the cloud environment. This study explores binary classification problems as pilots for real-time edge computing implementations. Images are resized to 128 × 128 × 3 pixels to facilitate the training process. To create a binary classifier with MobileNet, we replaced the original classification layer with a customized dense layer, using a ‘softmax’ activation function for one-hot encoding binary outputs. Additionally, we fine-tuned the model by experimenting with different parameter combinations. The data were split into training and validation sets using an 80–20% ratio. We assessed the trained model’s performance using accuracy, precision, recall, and F1 score metrics. Each model underwent training for 50 epochs.

### 3.3. Edge Environment Setup

In order to assemble the edge devices, the products involved and their corresponding functions in this case study are shown in Table 1. These setups mainly aim to incorporate a multimodal system, including visual, textual and audio, for on-site interactions. The edge device setup includes the following modules: Raspberry Pi 4, 8 GB Model B with 1.5 GHz 64-bit quad-core CPU (8 GB RAM); 32 GB Samsung EVO + Micro SD card (Class 10) preloaded with NOOBS; Arducam for Raspberry Pi Camera; Vilros 8-inch 1024 × 768 screen; Vilros 2.4 GHz mini wireless keyboard with touchpad mouse-USB receiver; Vilros mini Bluetooth speaker; Power Ridge portable power bank with AC outlet, 100 W 26,270 mAh; and Coral USB Accelerator. The total price for such an edge device module is around USD 430.84.

Note that the Edge TPU (Tensor Processing Unit) is a specialized, application-specific integrated circuit (ASIC) designed to enhance ML tasks, particularly deep learning inference, at the network’s edge [104]. The Edge TPU is optimized for efficiently running neural network models, with an architecture streamlined for operations common in ML, such as matrix multiplications and convolutions. In this research, deploying the quantized model on the Edge TPU involves several straightforward steps. First, the quantized TF Lite model (.tflite file) is further converted into an Edge TPU-compatible version using Google’s Edge TPU Compiler. Then, the compiler is run on the quantized model to generate a compiled version optimized for the Edge TPU (e.g., model_edgetpu.tflite). Second, the Edge TPU runtime library is installed on the Raspberry Pi’s operating system (Raspbian OS). Third, the Google Coral USB Accelerator, which contains the Edge TPU, is connected to the Raspberry Pi via a USB port. Finally, we should use this Edge TPU model to run inference and delegate these inference tasks to the Edge TPU hardware in Google Coral’s coding framework. In this configuration, the Raspberry Pi acts as a coordinator, preparing data and handling peripheral tasks, while the actual inference computation is offloaded to the Edge TPU. This approach leverages the specialized hardware acceleration capabilities of the Edge TPU, resulting in significant performance improvements for machine learning inference tasks compared to executing them solely on the Raspberry Pi’s CPU.

The required software modules in the Raspberry Pi environment include Raspbian OS (version 2.9.3.1) (operating system), TFLite (version 2.10.0), OpenCV (version 4.9.0) (vision), and pyttsx3 (version 2.90) (text-to-speech) modules. We prepared a Python script to leverage the PiCamera to capture stream images and to resize every image frame to 128 × 128 × 3 pixels. The quantized model can be hard copied to the Raspberry Pi through a USB driver. The script can run the quantized classification model to infer real-time images, and the classification results will be output as textual and audio formats. Audio broadcasts will be generated based on aggregated results from 15 consecutive frames classifications. The final product can be implemented offline with full portability and no latency.

## 4. Case Study

### 4.1. Material Classification

Automatic and real-time material classification is crucial for construction projects as it ensures accurate material usage, minimizes errors, and enables immediate adjustments, leading to cost savings and timely project completion. In this section, the research explores the deployment of a TFLite binary classification model on a Raspberry Pi to distinguish between the plywood and oriented strand board (OSB).

The data collection process was conducted within a controlled laboratory environment, where images of plywood and OSB were randomly captured from various angles. These raw video recordings were meticulously dissected into individual frames, resulting in a sizeable dataset. To enhance the diversity of the training set, data augmentation techniques such as flipping, shearing, and rotating were applied to each frame. As a result, the dataset was expanded to encompass a total of 7879 training images for plywood and 7900 for OSB, ensuring a rich and varied source of visual information for the model. For testing, a separate collection of images was obtained at different times using a different smartphone. This yielded a total of 259 test images for plywood and 322 for OSB, which were kept distinct from the training data to assess the model’s generalization capabilities effectively. Future work can also expand into more material categories to develop a multi-object classification system.

**Figure 5:**
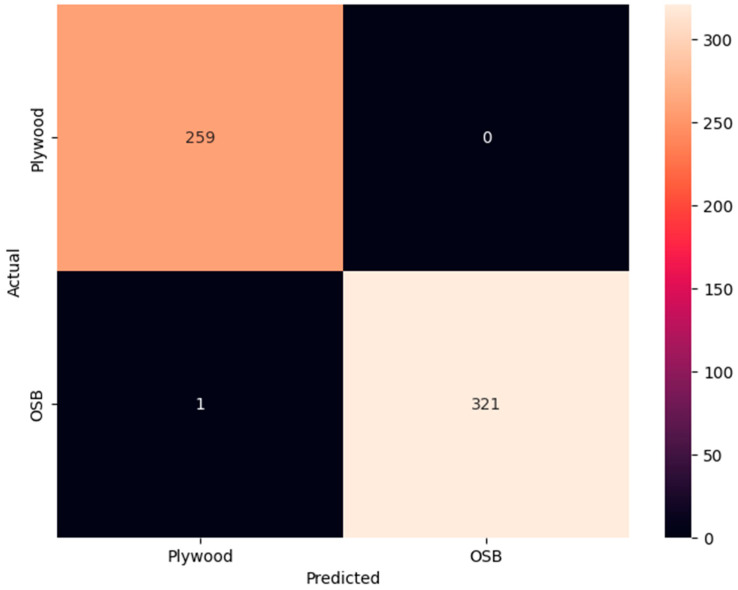
Confusion matrix of trained MobileNetV2 on the material classification task.

Table 2 displays the model performance statistics for the binary material classification task. Our comparison of various model versions revealed that both MobileNetV1 and MobileNetV2 achieved perfect accuracy. However, MobileNetV2 had a smaller model size. MobileNetV3Small, characterized by fewer parameters and the smallest model size among the tested models, achieved an accuracy of 0.91. Conversely, MobileNetV3Large, despite its larger model size, did not enhance overall performance. Considering the trade-offs between model size and performance accuracy, this study will use MobileNetV2 for further quantization. Figure 5 shows the confusion matrix for MobileNetV2 on the material classification task. The results show that, when the model predicts that the image is ‘Plywood’, only one image was misclassified as ‘OSB’, demonstrating promising results.

**Figure 6:**
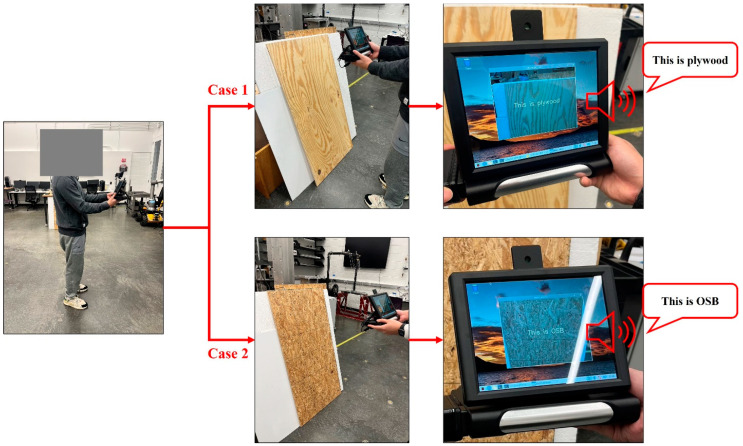
Material classification prototype implementation in the lab.

As a result of the quantization process, the original MbileNetV2 model, which had a size of 14.309 megabytes (MB), was efficiently compressed to a smaller size of 2.708 MB. This is approximately 19% of the original model size. Surprisingly, the accuracy, precision, recall, and F1 score still all approach 1. Figure 6 demonstrates the final implementation scenario. The implementation of the developed prototype is entirely portable, exhibits zero latency, operates offline, and offers real-time functionality, validating the effectiveness and robustness of the proposed framework. The identified materials would be displayed on the screen and broadcasted through the Bluetooth speaker.

### 4.2. Safety Detection: Identifying Boards with Nails

Poor construction housekeeping can jeopardize the safety of construction workers [10] and accounts for 15% of workplace deaths [105]. The Occupational Safety and Health Administration (OSHA) specifically states that any work areas, passageways, and stairs should be free of boards with protruding nails [106]. Therefore, this section will deploy an edge computing prototype to enable real-time safety detection to identify the construction boards with nails.

The data collected in the previous case can form a ‘boards’ dataset. In this round, we manually hammered nails into these boards in accordance with American Plywood Association [107] guidelines (regarding the locations and distance between nails, etc.) to form a ‘boards with nails’ dataset. Similarly, we video-recorded these boards from different angles. We then used another mobile device to collect test datasets. After data augmentation, 32,237 training data points emerged, with 16,779 images categorized as the ‘boards’ dataset and 15,458 images categorized as the ‘boards with nails’ dataset. Also, 1053 test datasets were formed, with 581 ‘boards’ and 472 ‘boards with nails’. These images were resized to 128 × 128 × 3 pixels for deployment efficiency. Note that during data collection, boards containing only one or two nails were classified as “boards with nails”. The researchers manually hammered each nail into the boards, capturing photographs progressively. Consequently, the “boards with nails” dataset comprises images with dynamic numbers of nails, documenting the entire process from boards with no nails to those fully equipped with all nails.

Table 3 presents the model performance statistics of different models on nail safety detection tasks. Based on a comparison of different model versions, we found that MobileNetV1 could return the highest accuracy value of around 0.90, with a 17.4 MB model size. MobileNetV2 showed the second highest accuracy value of around 0.87, with a 15.1 MB model size. MobileNetV3Small, with fewer model parameters, returned the lowest accuracy results. MobileNetV3Large, with the most parameters, also did not improve model accuracy. Moreover, the model must be sensitive to the presence of nails in the image. As a result, the MobileNetV1 was selected for further processing.

**Figure 7:**
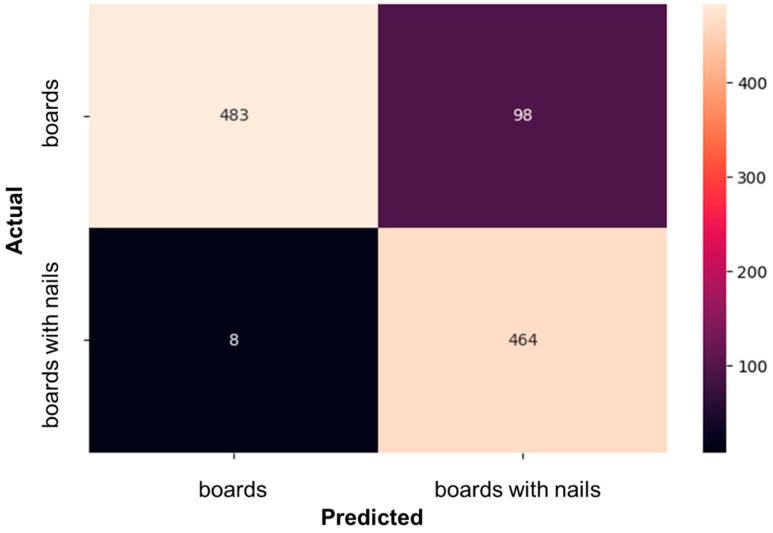
Confusion matrix of trained MobileNetV1 on the nail detection task.

Figure 7 shows the confusion matrix for MobileNetV1. The results show that when the model predicts that the image is ‘boards’, only eight images are misclassified as ‘boards with nails’. When the model predicts that the image is ‘boards with nails’, 98 images are misclassified as ‘boards’. Therefore, because the model is sensitive to the presence of nails, some suspicious board images are misclassified as ‘boards with nails’. Overall, however, MobileNetV1 demonstrates promising results, which we intend to leverage for further development.

After quantization, the MobileNetV1 model was reduced to 3.48 MB, approximately 20% of its original size. Remarkably, the quantized model maintained an accuracy of 0.9 in detecting nails in boards, closely matching the performance of the original. Other quantized models also showed similar accuracy to their original versions, with discrepancies appearing only from the third decimal place onward. This outcome aligns with the goals of TFLite Lite quantization [108]. Overall, the 3.48 MB quantized MobileNetV1 model is notably compact for an ML model, making it especially suitable for edge devices with limited storage capacity. This model will be deployed for real-time inference, where the ‘boards’ results will not broadcast any sound, and the ‘boards with nails’ will broadcast “Pay attention! There are nails in the boards”.

**Figure 8:**
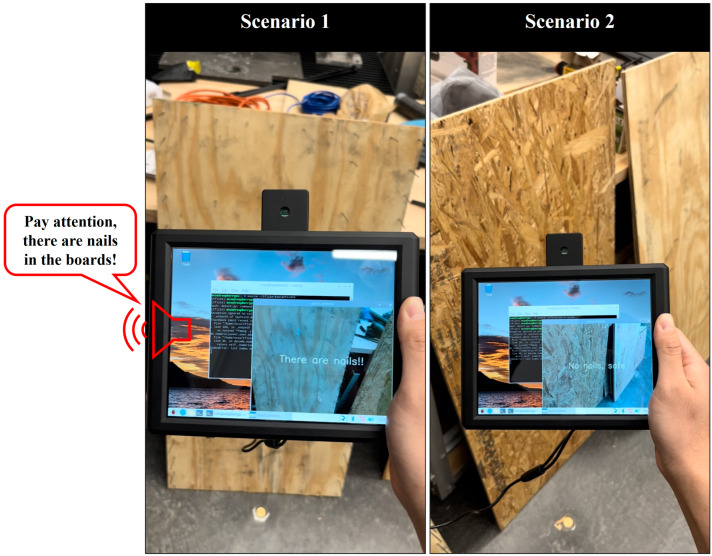
Real-time edge computing prototype implementation in the lab environment: Scenario 1 is the detection of a “board with nails”; Scenario 2 is the detection of a “board”.

Figure 8 shows a screenshot of the real-time implementation in the lab. The prototype was not connected to any cloud or internet services. Scenario 1 shows “There are nails” on the screen when the prototype is directly facing the “board with nails” object, and the prototype makes the alert of “pay attention, there are nails in the boards”. Scenario 2 shows “No nails, safe” on the screen when the prototype moves to target the “board” object; this case will not make a safety alert. The prototype demonstrated zero latency when predicting every framework of a real-time video stream. Furthermore, the prototype is fully portable and offline and can process data in a fully decentralized way without having to send data to a central server, thereby offering real-time decision-making advantages and cost-effective bandwidth usage. A video showing the implementation of the edge computing prototype can be found at the end of the paper.

## 5. Limitations

**Figure 9:**
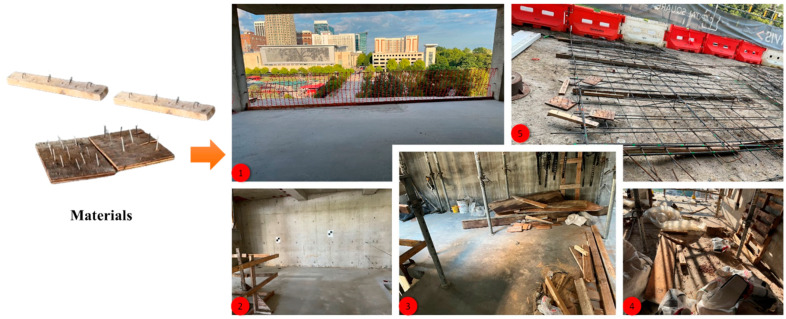
Experimental setup at a real construction site: Location 1 is an image taken from inside the building under construction showing downtown Raleigh, NC; Location 2 shows an interior room without scattered materials; Location 3 shows scattered building materials; Location 4 shows cluttered construction materials and debris; and Location 5 shows grid and buffer materials on the grounds of the building site.

In order to test the scalability and adaptability of the prototype for a real construction site scenario, we took the nail safety detection prototype to a construction site in Raleigh, North Carolina, where the internet was not accessible. As illustrated in the images presented in Figure 9, we placed numerous boards and beam timbers with intruded nails in five different locations in the building under construction and tested the prototype’s adaptability. The materials used in this stage of the validation process were not included in any of the model training. We found that the prototype performed adequately at these actual construction site locations but also showed extensive room for improvement. A robust implementation of the prototype could be impeded for several reasons, such as unseen datasets, insufficient lighting, and a chaotic construction environment. Furthermore, Figure 9 also shows that multiple construction locations (Locations 3, 4, and 5) were full of scattered building materials, which added noise to the model predictions. In future research, we will consider improving the prototype’s scalability and adaptability based on these considerations.

## 6. Recommendations for Future Research

The case study involved training the model in cloud environments and performing model inference entirely on a local device. This complies with level 4 edge intelligence (EI), according to Zhou et al. [26]. They identified a six-level rating for EI, with Level 6 representing scenarios where both training and inference occur entirely on the device, and Level 1 is all on the cloud. Currently, training small-scale or specialized models on edge devices is feasible if optimized algorithms and model compression techniques are seamlessly integrated, enabling more adaptive real-time model parameter adjustments. However, training large or complex models directly on edge devices is impractical due to constraints in computational resources, memory, and energy. The continuous advancements of microchips provide potential opportunities to fully create data, train models, and run inference solely on the edge. However, it does not necessarily mean all devices are at the “best level”. Instead, future cloud-edge coordination should consider multicriteria, such as latency, data security, energy efficiency, and hardware cost. One possibility is to embed microchips in edge devices and integrate federated learning, allowing each edge node to collect, process, and train its own data while preserving data privacy [109]. This enables parallel data computing to adjust model parameters, resulting in more reliable decision-making.

Integrating the Edge TPU can further enhance performance by offering high efficiency, low latency, and low power consumption. This is especially advantageous for running the modern open source large language models (LLMs, e.g., LlaMa, Mistral) on edge devices, which require significant computational resources. The rise of these large-scale models indicates shifting computational loads from the training phase to inference, where inference may emerge as a new bottleneck if the deployment process remains outdated. Integrating edge computing and LLM can enable an edge “brain” to perform more complicated tasks. Future research could explore developing a framework that enables intelligent, knowledge-driven edge “assistant” powered by LLMs on edge devices. This would allow for local utilization to better support engineers in their daily tasks.

Lastly, the integration of edge computing and robotics in the construction industry can potentially revolutionize traditional practices. Edge computing’s ability to process data locally and in real time enhances the responsiveness and autonomy of robotics. On the contrary, robotics provide autonomous mobility that can enable remote project operation. They also generate valuable data that can be locally processed, providing insights for efficient project management. Robotics benefit from immediate access to processed data, simulating various “what-if” cases, enabling quicker decision-making, improved navigation, and the ability to adapt to changing conditions in real time.

## 7. Conclusions

As the construction industry increasingly harnesses the power of image-based AI models for real-time monitoring, inspection, and tracking, ensuring seamless deployment of these applications on construction sites has become essential, especially in on-site locations with limited access to high-speed internet. This research introduces a comprehensive edge computing framework tailored for two practical applications: material classification and safety detection. By leveraging the TL MobileNet model, a binary image classification system was developed and subsequently quantized using TFLite to optimize performance. To facilitate real-time ML tasks, the researchers equipped Raspberry Pi edge devices with a battery, camera, audio module, and Edge TPU hardware. The Raspberry Pi environment was further enhanced with vision and text-to-audio modules, streamlining the deployment process. The results showed that (1) both case studies demonstrated high accuracy in classification tasks; (2) the quantized models were approximately 20% of their original sizes while maintaining the same accuracy; (3) the prototype achieved zero latency in differentiating materials and identifying hazardous nails without any internet connectivity; and (4) the system effectively integrated vision detection results with audio and textual outputs, promoting a multimodal synchronization application.

The contributions of this research are threefold. First, this study developed an efficient and generalized edge computing framework, with a focus on TF and quantization processes, to optimize real-time image classification on construction sites. This framework allows construction companies to perform any sophisticated image classifications on the edge without compromising model accuracy or requiring additional investment in computing resources, paving the way for future AI applications on construction sites. Second, a multimodal synchronization mechanism was introduced that processes visual data inputs and outputs textual and audio data within an edge computing environment. This mechanism not only facilitates interactive communication among on-site collaborators but also enhances situational awareness, enabling more immediate decision-making. Lastly, this research provides practical examples that demonstrate the effectiveness and efficiency of the proposed framework, addressing a common gap in the industry. The study presents a comprehensive set of hardware and software modules, integrating all components seamlessly to demonstrate real-time use cases and their benefits for construction management. By adopting this prototype, project managers can reduce centralized management efforts and promote a human-technology interaction environment for future construction. Project managers can also envision a more intelligent edge “assistant” by adding additional modules based on the developed framework.

## Figures and Tables

**Table 1:** Involved products of the developed edge device.

Product	Function	Price (USD)
CanaKit Raspberry Pi 4 8 GB Starter Kit—8 GB RAM	Single Board of Computer	159.99
Arducam for Raspberry Pi Camera Module	Camera	12.99
Vilros 8 Inch 1024 × 768 Screen	Screen	79.99
Vilros 2.4 GHz Mini Wireless Keyboard with Touchpad Mouse-USB Receiver	Keyboard with Touchpad Mouse	14.99
Vilros Mini Bluetooth Speaker	Speaker	8.99
Power Ridge Portable Power Bank with AC Outlet, 100 W 26,270 mAh	Battery	69.99
Coral USB Accelerator	Google Edge TPU ML accelerator coprocessor	83.90
	SUM	430.84

**Table 2:** Performance statistics of different models in binary material classification.

Model	Accuracy	Class	Precision	Recall	F1 Score	Support	Model Size
MobileNetV1	1	Plywood	1.00	1.00	1.00	259	27.5 MB
		OSB	1.00	1.00	1.00	322	
MobileNetV2	1	Plywood	1.00	1.00	1.00	259	14.3 MB
		OSB	1.00	1.00	1.00	322	
MobileNetV3Small	0.91	Plywood	0.89	0.91	0.90	259	9.7 MB
		OSB	0.93	0.91	0.92	322	
MobileNetV3Large	0.89	Plywood	0.81	1.00	0.90	259	26.5 MB
		OSB	1.00	0.82	0.90	322	

**Table 3:** Performance statistics of different models in nail safety detection.

Model	Accuracy	Class	Precision	Recall	F1 Score	Support	Model Size
MobileNetV1	0.90	Boards	0.98	0.83	0.90	581	17.4 MB
		Boards with nails	0.83	0.98	0.90	472	
MobileNetV2	0.87	Boards	0.97	0.79	0.87	581	15.1 MB
		Boards with nails	0.79	0.97	0.87	472	
MobileNetV3Small	0.73	Boards	0.92	0.57	0.70	581	9.4 MB
		Boards with nails	0.64	0.94	0.76	472	
MobileNetV3Large	0.83	Boards	0.80	0.92	0.85	581	19.2 MB
		Boards with nails	0.88	0.71	0.79	472	

## Data Availability

The data presented in this study are available on request from the corresponding author.

## Supplementary Materials

The reviewed 106 papers can be found at: https://docs.google.com/spreadsheets/d/e/2PACX-1vTklHOvdLNyAyXTM62zXn85SByvPAoVrORclSvjNOBwmjqreDNZ4ehXvVBfCfTFrQ/pubhtml (accessed on 24 November 2023). The real-time edge computing-enabled safety detection demo can be found at: https://www.youtube.com/watch?v=jtZXUE13TmE (accessed on 24 November 2023).
